# Validation of immunochromatographic test in broth-enriched rectal swab specimens

**DOI:** 10.1016/j.bjid.2025.104559

**Published:** 2025-06-30

**Authors:** Camila Loredana P.A.M. Bezerra, Barbara de Almeida Lessa Castro, Camila Rizek, Sania Alves dos Santos, Juliana Januário Gaudereto, Ana Paula Cury, Natashia Reese, Fernanda C. Lessa, Susan Bollinger, Matias Chiarastelli Salomão, ICT validation Study Group

**Affiliations:** a Universidade de São Paulo, Faculdade de Medicina (FMUSP), Departamento de Doenças Infecciosas, São Paulo, SP, Brazil; b Universidade de São Paulo, Faculdade de Medicina (FMUSP), Departamento de Infectologia e Medicina Tropical, Laboratório de Bacteriologia (LIM-49), São Paulo, SP, Brazil; c Universidade de São Paulo, Faculdade de Medicina (FMUSP), Departamento de Patologia, Laboratório de Medicina Laboratorial (LIM-03), São Paulo, SP, Brazil; d Centers for Disease Control and Prevention, Division of Healthcare Quality Promotion, GA, , United States of America

**Keywords:** Immunochromatographic test, O.K.N.V.I. RESIST-5 ICT, Carbapenem-resistant enterobacterales, Carbapenemase, Fecal carriers, Intestinal colonization, Rectal swab

## Abstract

Rapid detection of Carbapenemase-Producing *Enterobacterales* (CPE) is essential for informing infection prevention and control actions to curb the spread of antimicrobial resistance. Immunochromatographic Tests (ICTs) offer a quick and cost-effective alternative to molecular methods but are typically designed for bacterial isolates rather than direct clinical specimens. We developed a protocol using the O.K.N.V.I. RESIST-5 ICT (Coris Bioconcept, Gembloux, Belgium) to detect KPC, NDM, OXA-48, VIM, and IMP carbapenemases from broth-enriched mock rectal swabs. A total of 35 well-characterized carbapenemase-producing isolates were inoculated into a 10 % stool matrix to create mock swabs. Swabs were incubated in Brain-Heart Infusion (BHI) broth, with and without a 10 μg meropenem disk, at 37 °C for 4 and 6 h. After incubation, broths were centrifuged, and pellets were tested using the ICT. Sensitivity, specificity, and accuracy were calculated for each method (with/without meropenem disk and incubation period). Optimal performance was achieved with swabs incubated in BHI broth without meropenem for 6 h, showing 100 % sensitivity, specificity, and accuracy for all the five enzymes tested. Incubation with meropenem or shorter incubation times resulted in lower sensitivities, with per-enzyme sensitivities ranging from 0 % to 100 %. The developed protocol enables rapid and accurate detection of five common carbapenemases directly from broth-enriched rectal swabs within 6 h. This method offers a practical alternative to culture-based and molecular techniques, potentially enhancing infection control measures through timely identification of CPE.

## Introduction

Antimicrobial Resistance (AMR) has emerged as a major global public health problem, posing challenges in the treatment of community- and healthcare-acquired infections. This resistance leads to longer hospital stays, increased morbidity, mortality, and costs.^1^^,^^2^ The emergence of Extended-Spectrum β-Lactamases (ESBLs) in the 1980s has resulted in increased use of carbapenems, which in consequence led to the emergence of carbapenemases in the 1990s. *Klebsiella Pneumoniae* Carbapenemase (KPC) was the first enzyme capable of hydrolyzing carbapenems to be described, followed by other enzymes with similar hydrolysis capacity such as New Delhi Metallo beta-lactamase (NDM), Oxacillinase-48 (OXA-48), Verona integron-encoded MBL (VIM), and Imipenemase (IMP), which have been identified in Gram-negative bacteria found in healthcare settings.^3^, ^4^, ^5^ The U.S. Centers for Disease Control and Prevention (CDC) has identified carbapenem-resistant *Enterobacterales* (CRE) as an urgent threat to human health in 2019 and in 2024 WHO published a bacterial pathogen priority list where CRE is listed as a critical AMR public health threat.^6^^,^^7^

Control strategies such as implementing Contact Precautions are based on identifying and isolating patients infected or colonized with Carbapenemase-Producing Enterobacterales (CPE) and other Carbapenemase-Producing Organisms (CPO).^8^^,^^9^ These strategies have been relatively effective but rely on rapid and accurate laboratory detection of carbapenemase production. Colonization screening strategies often use culture-based methods, which are laborious and time-consuming and only detect carbapenem resistance. Subsequent phenotypic assays can detect carbapenemase production in isolates but fail to identify the specific carbapenemase present which are important to monitor emergence of resistance genes and inform response strategies. Culture-independent molecular assays using Polymerase Chain Reaction (PCR) can rapidly detect and identify specific carbapenemase genes directly from rectal swabs, but are more expensive and less commonly used, especially in Low- and Middle-Income Countries (LMIC).^3^^,^^10^

Immunochromatographic Tests (ICTs) are rapid tests capable of detecting and identifying carbapenemase enzymes through lateral flow antigen-antibody reaction. They are easy to perform, less expensive than molecular methods, and demonstrate good sensitivity and specificity.^3^ ICTs were designed and approved for use with bacterial isolates, but studies have shown good performance when testing broth-enriched rectal swabs, which reduces the turnaround time compared to traditional culture.^11^^,^^12^

This study describes the development and validation of a protocol for detecting KPC, NDM, OXA-48, VIM, and IMP carbapenemases in broth-enriched mock rectal swabs using the O.K.N.V.I. RESIST-5 ICT (Coris Bioconcept, Gembloux, Belgium) at Hospital das Clinicas of the University of Sao Paulo, Brazil. By offering a simple, rapid, and efficient screening approach, this protocol can help control the spread of CPE and other CPO in healthcare settings.

## Materials and methods

### Verification of ICT performance using pure isolates

Prior to experimenting with broth enrichment, we needed to confirm that the kit would perform as expected when testing pure isolates. We verified the O.K.N. and V.I. cassettes (“K-set”) within the O.K.N.V.I. RESIST-5 ICT kit for the detection of OXA-48, KPC, NDM, and VIM, and IMP carbapenemases, respectively, using well-characterized isolates of CRE from the Medical Investigation Laboratory 49 (LIM-49) collection and from the CDC & FDA Antimicrobial Resistance Isolate Bank.^13^ These well-characterized isolates were verified for purity and identified using matrix-assisted laser desorption-ionization time of flight mass spectrometry (MALDI-TOF MS) and 16S rRNA gene sequencing (as needed). Antimicrobial Susceptibility Testing (AST) was performed using the reference broth microdilution method in accordance with Clinical and Laboratory Standards Institute (CLSI) M100-S23 standard.^14^ In addition, whole genome sequencing was performed on isolates to identify resistance markers and to better understand the genotypic basis of resistance. We tested 70 CRE isolates, including 57 producing one or more of the five targeted carbapenemases [KPC (15), NDM (15), OXA-48 (12), IMP (5), VIM (7), NDM/OXA-48 dual producer (3)], and 13 non-carbapenemase producing CRE (Supplementary Table 1).

To prepare the isolates for testing, we grew them on a MacConkey agar plate using a sterile loop and then incubated them at 37 °C for 18‒24 h. After a growth and purity check, a subculture was prepared with a meropenem disk and incubated at 37 °C for 18‒24 h to ensure that the isolates fully expressed all traits prior to ICT testing. From the subculture, with a sterile loop, we took a colony and mixed it with 30 µL of lysis buffer C and 8 drops of lysis buffer A. A total of 100 µL of this suspension were added to each sample port of the O.K.N. and V.I. K-sets. Results were read after 15 min, according to the manufacturer’s instructions. The ICT results were compared with the expected results from previous Whole-Genome Sequencing (WGS) characterization by LIM-49 or the CDC and FDA AR Isolate Bank. Quality control was performed daily and included a negative and a positive control for each carbapenemase tested.

### Optimization and validation of a protocol for ICT performance using broth-enriched mock rectal swabs spiked with carbapenem-resistant isolates

In this step, we created mock rectal swabs inoculated with well-characterized isolates and incubated the swabs in BHI broths, with and without meropenem, for 4 and 6 h, then tested aliquots of the broths at each time point using ICT. The stool matrix was created by pooling clinical specimen remnants from five different donors for whom Fecal Occult Blood Test (FOBT) results were negative. Prior to pooling, we confirmed with culture and carbapenem disk diffusion, GeneXpert Carba R (Cepheid, Sunnyvale, CA, USA), and O.K.N.V.I. RESIST-5 that all stools were negative for carbapenem-resistant bacteria and negative for the 5-carbapenemase genes (bla_KPC_, bla_OXA-48_, bla_NDM_, bla_IMP_, and bla_VIM_). Pooled stool was diluted to a 10 % concentration using sterile saline and divided into 50 µL aliquots.

Of the 70 well-characterized CRE isolates from LIM-49 and the CDC & FDA AR Isolate Bank, 35-carbapenemase producing CREs were selected for the protocol optimization process and prepared as described above, including 11 KPC, 5 OXA-48, 1 OXA-48-like, 6 NDM, 4 IMP, 5 VIM, 3 NDM/OXA-48. For each isolate, we made a saline suspension of 1.5 × 10^6^ CFU/mL. We found this to be an optimal concentration after testing the limit of detection of a KPC-producing *Klebsiella pneumoniae* isolate at different inoculation sizes and incubation times.

We then combined 150 µL of each isolate suspension with 50 µL of the 10 % stool matrix in a microfuge tube, to a final concentration of 1.125 × 10^6^ CFU/mL, and immersed two swabs (Copan with Stuart medium), one at a time, until the entire swab was covered. For seven isolates, each swab was inserted into gel Stuart's medium prior to testing to simulate transport conditions for rectal swabs from real patients.

Our initial methodology for testing the mock rectal swabs involved incubation in thioglycolate broth, with and without meropenem, for 4 h, 6 hours, and 24 h. However, during pilot testing, we encountered a large number of false positive and false negative results that required modifications to our initial protocol (Supplementary Table 2).

The modified and final protocol consisted of incubating one swab in 10 mL of Brain-Heart Infusion (BHI) broth with a 10 μg meropenem disk and another swab in 10 mL of BHI without meropenem, both for 4 h and 6 h at 37 °C.

At each time point, we centrifuged 2 mL aliquots of the enrichment broth for 5 min at 11,000 × *g*. The supernatant was discarded, the pellet was mixed with 30 µL of lysis buffer C and 8 drops of lysis buffer A. A total of 100 µL of this suspension were added to the O.K.N. and V.I. K-sets. Results were read after 15 min by two independent observers. The result was considered positive when the control band and at least one carbapenemase-enzyme band were visible and negative if only the control band was visible.

### Establishing limit of detection

Limit of Detection (LOD) testing was performed by creating mock rectal swabs each spiked with a well-characterized CPE isolate for each gene target of interest (KPC, NDM, OXA-48-like, VIM, and NDM/OXA-48-like). A 0.5 McFarland was prepared for each isolate, diluted 10-fold down to 10^2^, then from each serial dilution, 300 µL was added to 100 µL 10 % negative stool matrix. A sterile Copan swab with Stuart media was saturated individually with each spiked stool solution and immediately placed in 10 mL BHI broth for 6 h at 37 °C in ambient air. After incubation, 2 mL of the BHI broth was centrifuged for 5 min at 11,000 × *g*. The pellet was resuspended in 30 µL of lysis buffer C and 8 drops of lysis Buffer A and added to each K-set according to the IFU. Testing was performed in duplicate across two days for a total of four replicates. The estimated LOD for each enzyme target was determined where detection frequency was observed to be 100 % across all replicates.

### Statistical analysis

Sensitivity, specificity, and accuracy were calculated for each enrichment time period and broth condition (± meropenem) combination: four hours without meropenem (4H), four hours with meropenem (4HM), six hours without meropenem (6H), and six hours with meropenem (6HM). We used the 35 sequenced isolates from the LIM-49 and the CDC & FDA AR Bank as gold standards to calculate the sensitivity, specificity, and accuracy of the ICT using the enriched broth. 95 % Confidence intervals were calculated using the Clopper-Pearson method, or Logit 95 %, when adequate. Statistical analysis was performed using STATA version 17 (StataCorp, College Station, Texas, USA).

### Ethical aspects

The study protocol was reviewed and approved by the Institutional Review Boards of Hospital das Clínicas (CAAE 47,011,921.9.0000.0068), the CDC (0900f3eb8208d8e8), and Johns Hopkins (20,294).

## Results

When testing pure colonies, all ICT results agreed with the expected results except for one false negative from an NDM-producing *Enterobacter cloacae* isolate. After repeat testing of the same isolate grown on blood agar instead of MacConkey, the result was positive for the NDM enzyme for an overall agreement of 100 %.

When testing broth aliquots from the spiked mock rectal swabs, the best performance was seen with swabs incubated in BHI without meropenem for 6 h. This combination demonstrated sensitivity, specificity, and accuracy of 100 % for each enzyme tested. For all other combinations, the ICT demonstrated 100 % specificity, showing no false positive results for any of the enzymes in either incubation period, with or without meropenem. However, per-enzyme sensitivity varied greatly across the different combinations, ranging from 78 % to 100 % for OXA-48, 55 % to 100 % for KPC, 67 % to 100 % for NDM, 40 % to 100 % for VIM, and 0 % to 100 % for IMP, and ranging from 66 % to 100 % for the O.K.N. K-set and from 22 % to 100 % for the V. I. K-set (Table 1).

**Table 1:** Performance of O.K.N.V.I. RESIST-5 immunochromatographic test with broth-enriched mock rectal swabs compared to sequenced isolates.

Carbapenemase (n)	4 h of incubation with meropenem		
	Sensitivity % (95 % IC)	Specificity % (95 % IC)	Accuracy (95 % IC)
Overall (35)	51 % (34–69)	‒	‒
O.K.N. K-set (26)	66 % (46–82)	100 % (82–100)	89 % (80–94)
V.I. K-set (9)	22 % (3–60)	100 % (66–100)	61 % (36–83)
OXA-48 (9)	78 % (40–97)	100 % (83–100)	93 % (77–99)
KPC (11)	55 % (23–83)	100 % (81–100)	83 % (64–94)
NDM (9)	67 % (30–93)	100 % (83–100)	90 % (54–100)
VIM (5)	40 % (5–85)	100 % (40–100)	67 % (30–93)
IMP (4)	0 % (0–60)	100 % (48–100)	56 % (21–86)

	4 h of incubation without meropenem		
	Sensitivity	Specificity	Accuracy
Overall (35)	82 % (66–92)	–	–
O.K.N. K-set (26)	86 % (67–96)	100 % (86–100)	95 % (89–99)
V.I. K-set (9)	67 % (30–93)	100 % (66–100)	83 % (59–96)
OXA-48 (9)	89 % (52–100)	100 % (83–100)	97 % (83–100)
KPC (11)	91 % (59–100)	100 % (82–100)	97 % (82–100)
NDM (9)	78 % (40–97)	100 % (83–100)	93 % (77–99)
VIM (5)	80 % (28–99)	100 % (40–100)	89 % (52–100)
IMP (4)	50 % (7–93)	100 % (48–100)	78 % (40–97)

	6 h of incubation with meropenem		
	Sensitivity	Specificity	Accuracy
Overall (35)	82 % (66–92)	–	–
O.K.N. K-set (26)	90 % (73–98)	100 % (87–100)	97 % (90–99)
V.I. K-set (9)	56 % (21–86)	100 % (66–100)	78 % (52–94)
OXA-48 (9)	100 % (66–100)	100 % (83–100)	100 % (88–100)
KPC (11)	73 % (39–94)	100 % (81–100)	90 % (73–98)
NDM (9)	100 % (66–100)	100 % (83–100)	100 % (88–100)
VIM (5)	80 % (28–99)	100 % (40–100)	89 % (52–100)
IMP (4)	25 % (1–81)	100 % (48–100)	67 % (30–93)

	6 h of incubation without meropenem		
	Sensitivity	Specificity	Accuracy
Overall (35)	100 % (90–100)	–	–
O.K.N. K-set (26)	100 % (88–100)	100 % (94–100)	100 % (96–100)
V.I. K-set (9)	100 % (66–100)	100 % (66–100)	100 % (81–100)
OXA-48 (9)	100 % (66–100)	100 % (83–100)	100 % (88–100)
KPC (11)	100 % (72–100)	100 % (81–100)	100 % (88–100)
NDM (9)	100 % (66–100)	100 % (83–100)	100 % (88–100)
VIM (5)	100 % (48–100)	100 % (40–100)	100 % (66–100)
IMP (4)	100 % (40–100)	100 % (48–100)	100 % (66–100)

OXA-48, Oxacillinase-48; KPC, *Klebsiella pneumoniae* carbapenemase; NDM, New Delhi metallo-β-lactamase; VIM, Verona Integron-encoded Metallo-β-lactamase; IMP, Imipenemase Metallo-β-lactamase. The comparison group was carbapenemase-producing Enterobacterales isolates well-characterized by whole genome sequencing.

Within each time period, broths incubated without meropenem demonstrated equivalent or better ICT performance than those incubated with meropenem. Extending the incubation period to 6 h also improved performance independent of meropenem presence or absence. The ICT performed poorly for IMP and VIM enzymes across all broth conditions, except for the 6 h incubation period without meropenem, which showed good results. The performance of the ICT on dual enzyme producers, NDM and OXA-48 producers (*n* = 3), was also investigated in this study. Concomitant detection of both enzymes was observed with 100 % sensitivity in the 6 h incubation period, regardless of the presence or absence of meropenem. During the 4 h incubation period, concomitant detection of both enzymes was only observed for one of the three isolates tested: one tested negative for both enzymes and one tested negative for NDM and positive for OXA-48. Presence or absence of meropenem did not impact results during the 4 h incubation. Each condition is described in Supplementary Table 1.

Seven swabs were inserted into Stuart's gel medium prior to broth incubation (4 KPC, 1 NDM, 1 OXA-48, 1 IMP). Five of the seven were positive under all conditions, one KPC was not detected in the 4HM condition and only the 6 h protocol without meropenem correctly identified the presence of the IMP enzyme.

The estimated overall LOD for the O.K.N.V.I. was 1.125 × 10^5^ CFU. Notably, the LOD for KPC and OXA-48 targets was lower at 1.125 × 10^4^ CFU compared to 1.125 × 10^5^ CFU for the other mechanisms.

Incubation with thioglycolate broth, with and without meropenem, per our initial protocol, led to thick pellets that were difficult to aspirate after centrifugation. This led us to transition to BHI. With both thioglycolate and BHI, incubation for 24 h led to 18 false positives (Supplementary Table 2). PCR testing confirmed the absence of targeted carbapenemase genes in false-positive samples. Broths incubated for 4 h and 6 h had a neutral pH, but after 24 h changed to an acidic pH (5–6), presumably due to accumulation of acidic byproducts from the stool matrix leading to false positive ICT results. These results led to the use of BHI without meropenem with incubation periods of four and six hours as described in the methods.

## Discussion

Our study had similar findings to two other studies that evaluated ICT testing following broth enrichment.^11^^,^^12^ Fauconnier et al. incubated 149 real rectal swabs in Trypticase soy broth containing 0.25 mg/L meropenem for 2.5 h then concentrated and tested the broth using O.K.N. K-Set (Coris Bioconcept). They reported an overall sensitivity of 96 % and specificity of 100 % for the detection of OXA-48, KPC and NDM enzymes. Gallah et al. incubated mock rectal swabs in selective carbapenemase enrichment broth (CProbeBE; Coris BioConcept) for 3 h, then concentrated and tested the broth using O.K.N.V. RESIST-4 ICT (Coris Bioconcept). They reported overall sensitivity of 97.7 % and specificity of 100 % for the detection of OXA-48-like enzymes, KPC, NDM and VIM. Although neither of these studies reached 100 % sensitivity, both achieved strong results in half the TAT compared to our study, demonstrating the effect that different types of enrichment broth, antibiotic concentration, and stool inoculum can have on the required incubation time.

Our study evaluated broth with and without the addition of a 10 µg meropenem disk for a final concentration of 1 mg/L. The presence of meropenem during broth enrichment was anticipated to suppress growth of carbapenem susceptible organisms, thereby enhancing growth of CPOs and stimulate carbapenemase production. Surprisingly, in our experiments, omission of meropenem during the incubation period yielded superior results despite conventional expectations. One theory to explain this unexpected outcome is that in the absence of meropenem, the growth of bacteria producing weak or low-level carbapenemases was not inhibited, allowing them to grow sufficiently during the shorter incubation period to be detected by the ICT, thereby improving the test’s sensitivity. In contrast, when meropenem was present, these bacteria may not have grown enough within the limited incubation time. In a study validating another ICT (NG-Test CARBA-5), the authors also found that both a longer incubation time (1 hour vs. 4 h) and not using antibiotics in the enrichment broth resulted in higher test sensitivity.^15^

However, extended incubation can lead to false-positive results as we have shown with 24 h incubation. Therefore, an incubation of 6 h per our results seems sufficient for detection of low-level or weak carbapenemases.

Unlike previous studies, ours included the V.I. K-Set for detection of IMP and VIM carbapenemases. Detection of IMP carbapenemases can be challenging due to the diversity of nucleotide and protein sequences of different variants.^16^ The manufacturers of the O.K.N.V.I. RESIST-5 ICT recommend that additional broth enrichment tests are performed, and multiple variants are tested. We tested two IMP-4 and two IMP-8 variants. Sensitivity was very poor (range: 0 % to 50 %) with incubation periods of 4 or 6 h with meropenem disk or 4-hours without meropenem. These sensitivities were much lower than the O.K.N. enzymes (86 % to 100 %) and the 6-hour incubation with meropenem which demonstrated 100 % sensitivity and specificity. We did not observe any interference with ICT performance related to Stuart’s transport gel.

This protocol has the potential to significantly reduce the Turnaround Time (TAT) for CPE colonization screening compared to culture-based methods (48‒72 h), allowing for rapid response to implement Infection Prevention and Control (IPC) measures for CPE colonized patients. This can be effective in controlling CPE transmission.^17^^,^^18^ There are still few studies on the impact of rapid tests on the therapy and outcomes of patients with CPE infection, with most of the evidence being based on laboratory studies or expert opinions.^19^

An additional advantage of ICT over culture is that ICT detects active O.K.N.V.I carbapenemase production and identifies the enzyme responsible. Knowing the mechanism of resistance can assist the IPC team with actions. For example, if the detected carbapenemase is uncommon in the local epidemiology, the IPC team in addition to placing the patient on Contact Precautions may decide to initiate additional response activities such as contact screening of other patients. Therefore, rapid detection of CPE and the type of carbapenemases can inform targeted IPC interventions. Direct molecular methods offer the same advantage but require costly equipment and consumables and require specific collection devices which can be difficult to obtain in some countries. In addition, molecular methods can detect genetic material but cannot determine whether the carbapenemase is being expressed. ICT is less expensive, confirms production of the enzyme, and is a more accessible option. However, a notable limitation of rapid tests (ICT and molecular tests) is the inability to detect carbapenemases not included in their target panel.

During our study, we encountered challenges that may help others. Thioglycolate broth formed thick pellets after centrifugation, making aspiration difficult. False positives, especially after prolonged incubation, led us to try BHI broth. Switching to BHI significantly improved performance, reducing false positives and negatives, particularly with shorter incubation without meropenem. Extended incubation (24 h) decreased pH to acidic levels due to stool byproducts, contributing to false-positive ICT results ‒ a phenomenon also reported with other ICTs like COVID-19 rapid tests.^20^ Our findings highlight that a 6-hour incubation without meropenem offers optimal sensitivity and specificity, eliminating false positives and improving test accuracy.

The study presents several limitations. The performance evaluation is confined to the gene variants tested and cannot be extrapolated to other variants. We evaluated only one dual-producer gene combination and among only 3 isolates. This limitation could impact the generalizability of the results, particularly in settings where combinations other than NDM/OXA-48-like are present. Additionally, the use of mock rectal swabs introduces a degree of artificiality to the experimental setup, potentially influencing the performance outcomes compared to real clinical samples. While in vitro models are valuable for initial assessments, extrapolating findings to real-world scenarios should be approached with caution. The absence of quantitative assessment of carbapenemase expression further limits the ability to correlate enzyme levels with test sensitivity, especially in the context of enrichment conditions. Moreover, the study's small sample size and wide confidence intervals further constrain the robustness of the results. These limitations underscore the need for larger-scale studies to validate the findings and enhance statistical confidence.

In this study, we developed a robust new protocol for ICT detection of five common carbapenemases (KPC, NDM, OXA-48, VIM, and IMP) directly from broth-enriched mock rectal swabs with rapid results and 100 % sensitivity and specificity after 6 h of incubation without meropenem. This protocol has potential advantages compared to culture-based methods, such as identification of specific carbapenemases, reduced TAT, and lower cost compared to currently available molecular methods. Evaluation of this protocol on actual patient samples is critical to ensure if the findings we observed will hold in clinical settings with the potential to generate rapid and cost-effective results.

## Funding

This work was supported by the U.S. Centers for Disease Control and Prevention and Jhpiego (SBA-22-SBA-032) and the Fundação de Amparo à Pesquisa do Estado de São Paulo (2024/07836-1).

## Disclaimer

The findings and conclusions in this report are those of the authors and do not necessarily represent the official position of the U.S. Centers for Disease Control and Prevention.

This work has been partially presented as a poster at the European Congress of Clinical Microbiology and Infectious Diseases 2024.

## Conflicts of interest

The authors declare no have conflicts of interest.

## Appendix Supplementary materials

Image, application 1
