# Supplementary material for: Validation of immunochromatographic test in broth-enriched rectal swab specimens
Source: Braz J Infect Dis. 2025 Jun 30;29(5):104559. doi: 10.1016/j.bjid.2025.104559 (PMC12268539; doi:10.1016/j.bjid.2025.104559)
Supplement: Supplementary file 1 [file mmc1.docx]

**BJID-D-25-00035_ Supplementary Material**

**Supplementary Table 1** Well-characterized CRE isolates from the Medical Investigation Laboratory-49 (LIM-49) and isolates from the CDC & FDA AR Isolate Bank collection for the detection of KPC, NDM, OXA-48, VIM, and IMP carbapenemases in suspensions with and without meropenem discs, with 4 hours and 6 hours of incubation, using the O.K.N.V.I. RESIST-5 ICT.

| **Organism** | **Carbapenemase variants tested** | **O.K.N.V.I. RESIST-5 ICT results in rectal mock swabs** | | | |
| --- | --- | --- | --- | --- | --- |
|  |  | **4h with meropenem** | **4h without meropenem** | **6h with meropenem** | **6h without meropenem** |
| *Enterobacter cloacae*^a^ | KPC-3 | + | + | + | + |
| *Enterobacter cloacae*^a,c^ | KPC-3 | + | + | + | + |
| *Enterobacter cloacae¹* | KPC-3 | + | + | + | + |
| *Kluyvera ascorbata*^a^ | KPC-3 | - | + | - | + |
| *Raoultella ornithinolytica*^a^ | KPC-3 | - | - | - | + |
| *Serratia marcescens*^a^ | KPC-3 | + | + | + | + |
| *Escherichia coli*^a^ | KPC-4 | - | + | + | + |
| *Enterobacter cloacae*^a^ | KPC-6 | NT | NT | NT | NT |
| *Proteus mirabilis*^a^ | KPC-6 | - | + | - | + |
| *Klebsiella pneumoniae*^a^ | KPC-9 | NT | NT | NT | NT |
| *Klebsiella pneumoniae*^a^ | KPC-11 | NT | NT | NT | NT |
| *Klebsiella pneumoniae*^b,c^ | KPC+ | + | + | + | + |
| *Klebsiella pneumoniae*^b,c^ | KPC+ | + | + | + | + |
| *Klebsiella pneumoniae*^b,c^ | KPC+ | - | + | + | + |
| *Serratia marcescens*^a^ | KPC+ | NT | NT | NT | NT |
| *Serratia marcescens*^a^ | KPC+ | NT | NT | NT | NT |
| *Enterobacter cloacae*^a^ | NDM-1 | + | + | + | + |
| *Enterobacter cloacae*^a^ | NDM-1 | + | + | + | + |
| *Enterobacter cloacae*^a,c^ | NDM-1 | + | + | + | + |
| *Escherichia coli*^a^ | NDM-1 | NT | NT | NT | NT |
| *Klebsiella pneumoniae*^a^ | NDM-1 | - | + | + | + |
| *Klebsiella pneumoniae*^a^ | NDM-1 | NT | NT | NT | NT |
| *Morganella morganii*^a^ | NDM-1 | NT | NT | NT | NT |
| *Proteus mirabilis*^a^ | NDM-1 | NT | NT | NT | NT |
| *Providencia rettgeri*^a^ | NDM-1 | NT | NT | NT | NT |
| *Salmonella Senftenberg*^a^ | NDM-1 | NT | NT | NT | NT |
| *Escherichia coli*^a^ | NDM-5 | + | + | + | + |
| *Escherichia coli*^a^ | NDM-5 | NT | NT | NT | NT |
| *Escherichia coli*^a^ | NDM-6 | NT | NT | NT | NT |
| *Escherichia coli*^a^ | NDM-7 | + | + | + | + |
| *Klebsiella pneumoniae*^a^ | NDM-7 | NT | NT | NT | NT |
| *Citrobacter freundii*^a^ | OXA-48+ | NT | NT | NT | NT |
| *Enterobacter aerogenes*^a^ | OXA-48+ | + | + | + | + |
| *Escherichia coli*^a^ | OXA-48+ | NT | NT | NT | NT |
| *Klebsiella pneumoniae*^a^ | OXA-48+ | NT | NT | NT | NT |
| *Klebsiella pneumoniae*^a^ | OXA-48+ | NT | NT | NT | NT |
| *Klebsiella pneumoniae*^a^ | OXA-48+ | NT | NT | NT | NT |
| *Klebsiella ozaenae*^a^ | OXA-181 | - | + | + | + |
| *Klebsiella pneumoniae*^a,c^ | OXA-181 | + | + | + | + |
| *Klebsiella pneumoniae*^a^ | OXA-181 | + | + | + | + |
| *Klebsiella pneumoniae*^a^ | OXA-181 | NT | NT | NT | NT |
| *Klebsiella pneumoniae*^a^ | OXA-181 | NT | NT | NT | NT |
| *Klebsiella pneumoniae*^a^ | OXA-232 | + | + | + | + |
| *Klebsiella pneumoniae*^a^ | OXA-232 | + | + | + | + |
| *Klebsiella pneumoniae*^a^ | NDM-1; OXA-232 | - ; - | - ; - | + ; + | + ; + |
| *Klebsiella pneumoniae*^a^ | NDM-1; OXA-232 | - ; + | - ; + | + ; + | + ; + |
| *Klebsiella pneumoniae*^a^ | NDM-5; OXA-232 | + ; + | + ; + | + ; + | + ; + |
| *Enterobacter aerogenes*^a^ | IMP-4 | - | + | + | + |
| *Klebsiella oxytoca*^a,c^ | IMP-4 | - | - | - | + |
| *Klebsiella pneumoniae*^a^ | IMP-4 | - | - | - | + |
| *Klebsiella pneumoniae*^a^ | IMP-4 | - | + | - | + |
| *Enterobacter cloacae*^a^ | IMP-8 | NT | NT | NT | NT |
| *Enterobacter cloacae*^a^ | VIM-1 | - | + | - | + |
| *Enterobacter cloacae*^a^ | VIM-1 | - | + | + | + |
| *Enterobacter cloacae*^a^ | VIM-1 | NT | NT | NT | NT |
| *Klebsiella pneumoniae*^a^ | VIM-1 | + | + | + | + |
| *Klebsiella pneumoniae*^a^ | VIM-1 | + | + | + | + |
| *Klebsiella pneumoniae*^a^ | VIM-27 | - | - | + | + |
| *Klebsiella pneumoniae*^a^ | VIM-27 | NT | NT | NT | NT |
| *Escherichia coli*^b^ | Negative | NT | NT | NT | NT |
| *Escherichia coli*^b^ | Negative | NT | NT | NT | NT |
| *Escherichia coli*^b^ | Negative | NT | NT | NT | NT |
| *Escherichia coli*^b^ | Negative | NT | NT | NT | NT |
| *Escherichia coli*^b^ | Negative | NT | NT | NT | NT |
| *Escherichia coli*^b^ | Negative | NT | NT | NT | NT |
| *Escherichia coli*^b^ | Negative | NT | NT | NT | NT |
| *Klebsiella pneumoniae*^b^ | Negative | NT | NT | NT | NT |
| *Klebsiella pneumoniae*^b^ | Negative | NT | NT | NT | NT |
| *Klebsiella pneumoniae*^b^ | Negative | NT | NT | NT | NT |
| *Klebsiella pneumoniae*^b^ | Negative | NT | NT | NT | NT |
| *Klebsiella pneumoniae*^b^ | Negative | NT | NT | NT | NT |
| *Klebsiella pneumoniae*^b^ | Negative | NT | NT | NT | NT |

^a^ CDC & FDA AR Bank; ^b^LIM-49; NT Not tested with mock rectal swabs; ^c^ Samples tested with the Stuart transport media.

**Supplementary Table 2** Tests on well characterized CDC & FDA AR Bank and LIM-49 resistant isolates KPC-, NDM-, IMP-, VIM- and OXA-48-producing isolates with thioglycolate and BHI; with stool matrix, with and without meropenem, and incubated for 4h, 6h and 24h.

|  | | | **O.K.N.V.I. RESIST-5 ICT results** | | | | | | | | | | | | | | |
| --- | --- | --- | --- | --- | --- | --- | --- | --- | --- | --- | --- | --- | --- | --- | --- | --- | --- |
|  |  |  | **KPC** | | | **OXA** | | | **IMP** | | | **VIM** | | | **NDM** | | |
|  |  |  | **4h** | **6h** | **24h** | **4h** | **6h** | **24h** | **4h** | **6h** | **24h** | **4h** | **6h** | **24h** | **4h** | **6h** | **24h** |
| ***Klebsiella pneumoniae* IMP+** | **BHI** | **With**  **Meropenem** | - | - | - | - | - | - | - | - | - | - | - | - | - | - | - |
|  |  | **Without Meropenem** | - | + | - | - | - | - | + | + | + | - | - | - | - | - | - |
|  | **Thioglycolate** | **With**  **Meropenem** | - | - | - | - | - | - | - | - | - | - | - | - | - | - | - |
|  |  | **Without Meropenem** | - | - | + | - | - | - | - | - | + | - | - | + | - | - | - |
| ***Enterobacter aerogenes***  **OXA-48+** | **BHI** | **With**  **Meropenem** | - | - | - | + | + | + | - | - | + | - | - | - | - | - | - |
|  |  | **Without Meropenem** | - | - | - | + | + | + | - | - | - | - | - | - | - | - | - |
|  | **Thioglycolate** | **With**  **Meropenem** | - | - | + | + | + | + | + | + | + | - | - | + | - | - | - |
|  |  | **Without Meropenem** | **NT** | **NT** | **NT** | **NT** | **NT** | **NT** | **NT** | **NT** | **NT** | **NT** | **NT** | **NT** | **NT** | **NT** | **NT** |
| ***Escherichia coli***  **KPC+** | **BHI** | **With**  **Meropenem** | + | + | + | - | - | - | - | - | - | - | - | - | - | - | - |
|  |  | **Without Meropenem** | + | + | + | - | - | - | - | - | - | - | - | - | - | - | - |
|  | **Thioglycolate** | **With**  **Meropenem** | + | + | + | - | - | + | + | + | + | - | - | + | - | - | - |
|  |  | **Without Meropenem** | **NT** | **NT** | **NT** | **NT** | **NT** | **NT** | **NT** | **NT** | **NT** | **NT** | **NT** | **NT** | **NT** | **NT** | **NT** |
| ***Enterobacter cloacae***  **KPC+** | **BHI** | **With**  **Meropenem** | + | + | + | - | - | - | - | - | - | - | - | - | - | - | - |
|  |  | **Without Meropenem** | + | + | + | - | - | - | - | - | - | - | - | - | - | - | - |
|  | **Thioglycolate** | **With**  **Meropenem** | NT | NT | + | NT | NT | - | NT | NT | + | NT | NT | + | NT | NT | - |
|  |  | **Without Meropenem** | NT | NT | + | NT | NT | - | NT | NT | + | NT | NT | + | NT | NT | - |
| ***Klebsiella pneumoniae***  **IMP+** | **BHI** | **With**  **Meropenem** | - | - | + | - | - | - | - | - | + | - | - | - | - | - | - |
|  |  | **Without Meropenem** | - | - | + | - | - | - | + | + | + | - | - | - | - | - | - |
| ***Klebsiella pneumoniae***  **NDM** | **BHI** | **With**  **Meropenem** | - | - | + | - | - | - | - | - | + | - | - | - | - | + | - |
|  |  | **Without Meropenem** | - | - | + | - | - | - | - | - | + | - | - | - | + | + | + |

| + | True Positive |
| --- | --- |
| - | True Negative |
| - | False Negative |
| + | False Positive |
| NT | Not tested |
